# mRNA-Seq Analysis of the *Pseudoperonospora cubensis* Transcriptome During Cucumber (*Cucumis sativus* L.) Infection

**DOI:** 10.1371/journal.pone.0035796

**Published:** 2012-04-24

**Authors:** Elizabeth A. Savory, Bishwo N. Adhikari, John P. Hamilton, Brieanne Vaillancourt, C. Robin Buell, Brad Day

**Affiliations:** 1 Department of Plant Pathology, Michigan State University, East Lansing, Michigan, United States of America; 2 Department of Plant Biology, Michigan State University, East Lansing, Michigan, United States of America; Technical University of Denmark, Denmark

## Abstract

*Pseudoperonospora cubensis*, an oomycete, is the causal agent of cucurbit downy mildew, and is responsible for significant losses on cucurbit crops worldwide. While other oomycete plant pathogens have been extensively studied at the molecular level, *Ps. cubensis* and the molecular basis of its interaction with cucurbit hosts has not been well examined. Here, we present the first large-scale global gene expression analysis of *Ps. cubensis* infection of a susceptible *Cucumis sativus* cultivar, ‘Vlaspik’, and identification of genes with putative roles in infection, growth, and pathogenicity. Using high throughput whole transcriptome sequencing, we captured differential expression of 2383 *Ps. cubensis* genes in sporangia and at 1, 2, 3, 4, 6, and 8 days post-inoculation (dpi). Additionally, comparison of *Ps. cubensis* expression profiles with expression profiles from an infection time course of the oomycete pathogen *Phytophthora infestans* on *Solanum tuberosum* revealed similarities in expression patterns of 1,576–6,806 orthologous genes suggesting a substantial degree of overlap in molecular events in virulence between the biotrophic *Ps. cubensis* and the hemi-biotrophic *P. infestans*. Co-expression analyses identified distinct modules of *Ps. cubensis* genes that were representative of early, intermediate, and late infection stages. Collectively, these expression data have advanced our understanding of key molecular and genetic events in the virulence of *Ps. cubensis* and thus, provides a foundation for identifying mechanism(s) by which to engineer or effect resistance in the host.

## Introduction

The phytopathogenic oomycete *Pseudoperonospora cubensis*, the causative agent of cucurbit downy mildew [1], [2], infects a wide range of cucurbits, including cucumber (*Cucumis sativus* L.), squash (*Cucurbita* spp.), and melon (*Cucumis melo* L.). As an obligate biotroph, *Ps. cubensis* is dependent on its host for both reproduction and dispersal, and as such, has evolved a highly specialized host range limited to members of the *Cucurbitaceae*. At present, downy mildew is the most important foliar disease of cucurbits, affecting cucurbit production throughout the world [1], [2]. Under favorable conditions, *Ps. cubensis* is capable of infecting and defoliating a field in less than two weeks, and as a result, is responsible for devastating economic losses. For more than 50 years, control of downy mildew on cucumber in the U.S. was maintained through genetic resistance; however, since 2004, the likely introduction of a new pathotype into U.S. pathogen populations has resulted in a loss of this resistance [1]. While minimal knowledge of the genetic variation within *Ps. cubensis* exists - specifically related to virulence, pathogenicity, and host specificity among physiological races - the genetic basis of these processes, and the underlying mechanism(s) associated with infection have not been elucidated [1], [2], [3], [4].

To date, analyses of the *Ps. cubensis*-*C. sativus* interaction have been limited to the identification of the aforementioned physiological races, and have largely focused on the utilization of variation in host specificity for the identification and classification of pathotypes [3], [5]. To this end, six physiological pathotypes, or races, have been identified within populations in the U.S., Israel, and Japan, as well as additional races throughout Europe [1], [2], [4]. In the U.S., increased disease pressure on cucumber production since 2004 is hypothesized to be the result of the introduction of a new, more virulent pathotype, capable of overcoming the downy mildew resistance gene *dm-1*, that has been widely incorporated into commercial cucumber varieties since the 1940's [6]. While genetic analyses such as Amplified Fragment Length Polymorphism have been used to differentiate these physiological races [4] and some effort has been made to refine the species within *Pseudoperonospora* [6], [7], there is limited information available about pathogenicity or virulence genes in *Ps. cubensis* or the molecular-genetic basis of resistance to this pathogen in the cucurbits.

Recent work generated the first sequence assembly of the *Ps. cubensis* genome and subsequent *in silico* analysis has identified candidate effector proteins that may have either virulence or avirulence roles in *Ps. cubensis* infection [8], [9]. Structurally, oomycete effector proteins display a modular organization, consisting of a N-terminal signal peptide, a conserved RXLR (*Arg-X-Leu-Arg*, where “X” is any amino acid) translocation motif, followed by a variable C-terminal effector domain [10]. In short, it is the function and activity of the variable C-terminal effector domain that drives the activity of these molecules [10], [11]. A set of 61 candidate effectors were identified in the first draft of the *Ps. cubensis* genome [9] and included a large class of variants with sequence similarity to the canonical RXLR motif. Specifically, the function of a QXLR-containing effector, designated *Pc*QNE, was characterized and shown to be a member of a large family of *Ps. cubensis* QXLR nuclear-localized effectors, which was up-regulated during infection of cucumber [9]. Additionally, internalization of *Pc*QNE into the host cell was shown to require the QXLR-EER motif, thereby establishing a basic functional homology with the well-characterized *Phytophthora* spp. effector proteins [9]. While this work serves as a substantial development in understanding the genetic basis for pathogenicity in *Ps. cubensis*, additional work is needed to identify and characterize additional effectors and other proteins involved in establishment of infection and pathogen proliferation.

The accessibility of oomycete pathogen genome sequences, combined with gene expression data from both pathogen and host throughout the course of infection, can serve as a basis for identification and curation of genes that may have important roles in both virulence and avirulence [12], [13], [14], [15]. To date, oomycete RXLR effectors have been demonstrated to suppress basal host resistance [16], [17], as well as to activate effector-triggered immunity (ETI) [18], [19], [20], [21]. In addition to the RXLR class, other cytoplasmically-localized effectors have been identified in *Phytophthora* spp. [11]. The Crinkler (CRN) family, for example, has a conserved LXLFLAK motif necessary for translocation into the host cytoplasm and subsequent import into plant nuclei where they elicit a rapid cell death response [22], [23]. Finally, oomycete effectors have also been shown to function within the host apoplast, including functions as enzyme inhibitors [24], [25], [26], [27], [28], small cysteine-rich proteins [22], [29], [30], the Nep1-like family of proteins [31], [32], and CBEL (Cellulose Binding, Elicitor, and Lectin-like) proteins [33], [34].

The initial stages of pathogen infection of a plant host involve adhesion, penetration, and invasive growth within the host cell tissue. As such, cell wall degrading enzymes (CWDE), such as endoglucanases, cutinases, cellulases, and β-glucanases have evolved as essential components of an oomycete's repertoire for cell wall penetration [20]. Numerous CWDE have been identified computationally from the genomic sequences of several plant pathogenic oomycetes, including *Phytophthora sojae*, *Phytophthora ramorum*, *Hyaloperonospora arabidopsidis*, and *Pythium ultimum* [30], [35], [36]. In *P. sojae*, members of the family 5 and family 12 endoglucanases have been shown to be up-regulated during early stages of infection [37], [38]. However, in *H. arabidopsidis*, which causes downy mildew of *Arabidopsis thaliana*, CWDE-encoding mRNAs are reduced [36]. This could indicate an adaptation in downy mildew pathogens for evasion of recognition by their host, as break-down products from plant cell wall components can function as elicitors of defense responses [39].

Recent advancements in sequencing technologies have led to an explosive growth in the analysis of *in planta*-expressed genes of biotrophic plant pathogens [12], [40], [41], [42], [43], [44]. In the current study, we present the first global gene expression analysis of the infection stages of cucumber by the obligate oomycete pathogen *Ps. cubensis*, the causal agent of cucurbit downy mildew. Through the analysis of a susceptible cucumber cultivar interaction, we describe the identification of genes with putative roles in infection, growth and pathogenicity. Using next-generation sequencing technology, we assessed gene expression in *Ps. cubensis* in sporangia and at six time points of infection. By combining visual assessment of symptoms with light microscopy to monitor infection stages as well as minimizing collection of non-inoculated tissues, we were able to capture expression of 7,821 *Ps. cubensis* genes ranging from 159 genes at 1 days post inoculation (dpi) to 7,698 at 8 dpi. In total, this work represents a comprehensive examination of the key infection stages of *Ps. cubensis* growth and development. In total, the work described herein provides a foundation for further dissection of genes relevant to virulence in this obligate phytopathogen.

## Results and Discussion

### Characterization and sampling of *Ps. cubensis* infection stages

**Figure 1:**
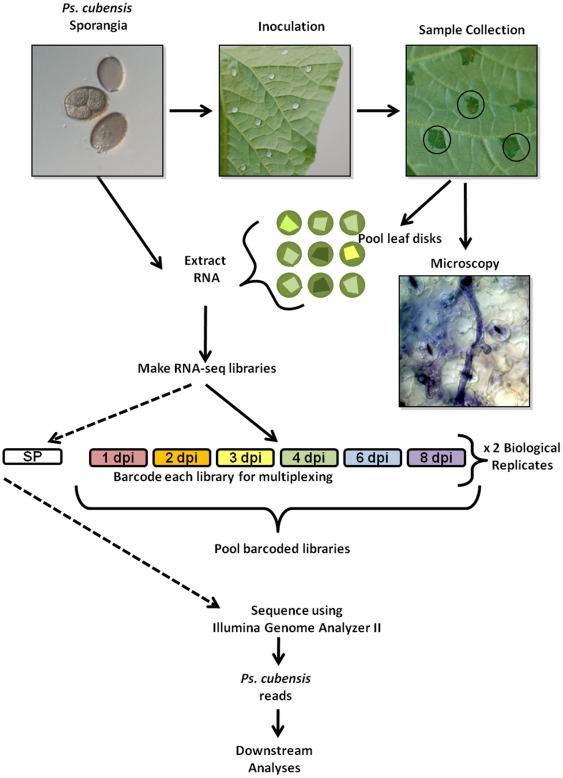
Experimental design and sample collection. A 1×10^5^ sporangia/ml solution of *Pseudoperonospora cubensis* was used to inoculate the abaxial leaf surface of cucumber cultivar ‘Vlaspik’. Samples were collected using a #3 cork borer to minimize uninfected tissue (black circles) at 1, 2, 3, 4, 6, and 8 days post-inoculation (dpi). Leaf disks were used for microscopic analysis of infection stages or pooled for RNA extraction. mRNA-Seq libraries were made for each time point from 2 biological replicates. Within a biological replicate, libraries were barcoded and sequenced in multiple lanes. The sporangia-only library (SP) was not barcoded and was sequenced on its own.

While *Ps. cubensis* is a major pathogen of cucumber and other cucurbits, limited resources describing the infection process and/or virulence determinants of this obligate oomycete are available. In the current study, we sought to identify *Ps. cubensis* gene expression from both purified sporangia, as well as from a time course of infected cucumber tissues, representing a wide range of infection stages from 1 to 8 dpi. In total, our goal was to gain a broad perspective of *in planta* gene expression during infection of a susceptible cucumber host and to correlate this expression with the development of outwardly visible symptoms, as well as the development of microscopic pathogen infection structures. Like other phytopathogenic downy mildews and biotrophic fungi, *Ps. cubensis* is non-culturable, and proliferates and reproduces only on a susceptible cucurbit host. As with previously published reports on analyzing gene expression in biotrophic phytopathogens, optimization of sampling techniques is key to maximize pathogen tissue compared to host, particularly at early stages of infection (Figure 1) [12], [43], [44], [45].

**Figure 2:**
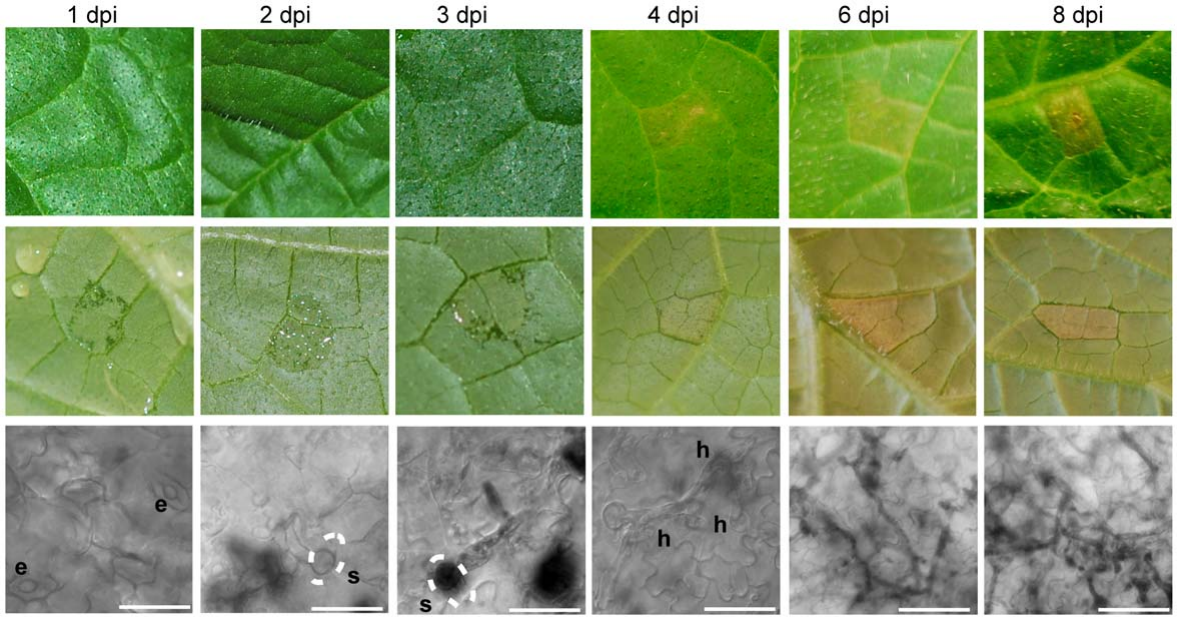
Symptoms and microscopy images of *Pseudoperonospora cubensis* infected *Cucumis sativus* cultivar ‘Vlaspik’ of time points used for transcriptome analysis. Symptom images were collected of the adaxial (top row) and abaxial (middle row) at 1, 2, 3, 4, 6, and 8 days post-inoculation (dpi). Microscopy (bottom row) to assess stages of *Ps. cubensis* invasion were collected from the same time points using ethanol-cleared, trypan blue stained samples. Scale bars at 1–4 dpi are 25 µm. Scale bars at 6 and 8 dpi are 50 µm. Dotted lines represent position of stomata relative to the pathogen structure. e = encysted zoospore. s = stomate. h = haustorium.

Plants were inoculated on the abaxial leaf surface with purified *Ps. cubensis* sporangia, and samples were collected using a cork borer, minimizing the amount of non-infected tissue in each sample (Figure 1). Initial symptoms of downy mildew infection can be observed on the abaxial leaf surface at 1–3 dpi as water soaking at the site of inoculation, while no visual symptoms are apparent on the upper leaf surface (Figure 2). At 1 dpi, zoospores were encysted upon stomata on the lower leaf surface, and by 2 dpi, appressoria and initial penetration hyphae were visible beneath stomata. The yellow angular lesions typical of cucurbit downy mildew were apparent on the upper leaf surface by 4 dpi, and over time, became more chlorotic and necrotic as the infection progressed. By 3 to 4 dpi, multiple haustoria formed within the mesophyll layer.

### mRNA-Seq data analyses

**Figure 3:**
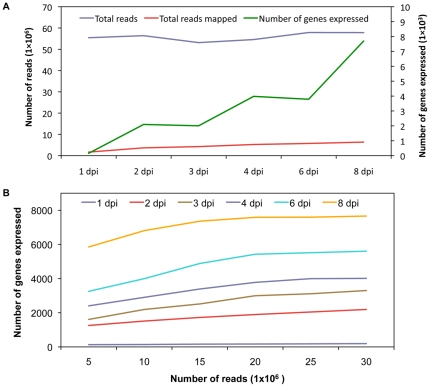
Number of total mRNA-Seq reads, reads mapped, and number of genes expressed. **A.** Total number of reads, number of reads mapped to the *Ps. cubensis* genome, and number of genes expressed by *Ps. cubensis* at different time points are shown. Reads were mapped using Bowtie version 0.12.5 [55] and TopHat version 1.2.0 [55]. Fragments per kilobase pair of exon model per million fragments mapped (FPKM) values were calculated using Cufflinks version 0.9.3 [56]. Genes were considered expressed if the FPKM and 95% confidence interval lower boundary FPKM value was greater than 0.001 and zero, respectively. dpi = days post-inoculation. **B.** Comparison between number of expressed genes detected and sampling depth. For all time points 5, 10, 15, 20, 25, and 30 million reads were randomly selected from the total pool of reads from different time points. Read mapping and expression abundances are as described in panel 3A.

Expression profiling of *Ps. cubensis* sporangia, as well as infection stages at six time points of cucumber infection, were performed using mRNA-Seq. For each time point, two biological replicates were sequenced. The total number of reads produced for each time point ranged from 55 to 59 million reads, with a median of 57 million reads. Reads were mapped to the *Ps. cubensis* genome which was generated by assembly of Illumina next generation reads; in total the *Ps. cubensis* genome encompasses 67.9 Mb, with 23,519 protein coding genes and 23,522 gene models [8]. Of the total reads generated, for each time point, approximately 1.6 to 6.4 million (3–12% of the total; Figure 3A) mapped to the *Ps. cubensis* genome. In turn, a majority of reads in each sample were of host origin, and mapped to the cucumber genome [46] (Figure S1). Through this analysis, we found that there was no significant difference in the total number of combined reads from different time points (p>0.80); however, the number of *Ps. cubensis* genes expressed at each time point was significantly different for all time point comparisons (p<0.05; Figure 3A). To assess the experimental variation attributable to biological variation, we compared the gene expression pattern of the genes expressed in both of our biological replicates. In total, our experiments showed very high levels of correlation for biological replicates (in all cases examined, Pearson's Correlation Coefficient (PCC) >0.94; Figure S2), indicating that our sampling, assay, and analysis methods are robust.

To evaluate the effect of sampling depth on gene expression detection and to assess whether we have adequately sampled the mixed mRNA-Seq read pool for *Ps. cubensis* transcripts, subsets of five to 30 million reads were randomly selected from the total read pool from each time point and mapped to the *Ps. cubensis* genome. The simulation experiment showed a clear positive relationship between sampling depth and number of expressed genes at the lower to medium sequencing depth (5 to 20 million reads; Figure 3B). With the exception of 1 dpi in which few genes are expressed, after 20–25 million reads, the number of expressed genes begin to plateau, corresponding to the minimum sampling depth of all libraries sequenced in this study.

### mRNA-Seq transcriptome profiles

**Figure 4:**
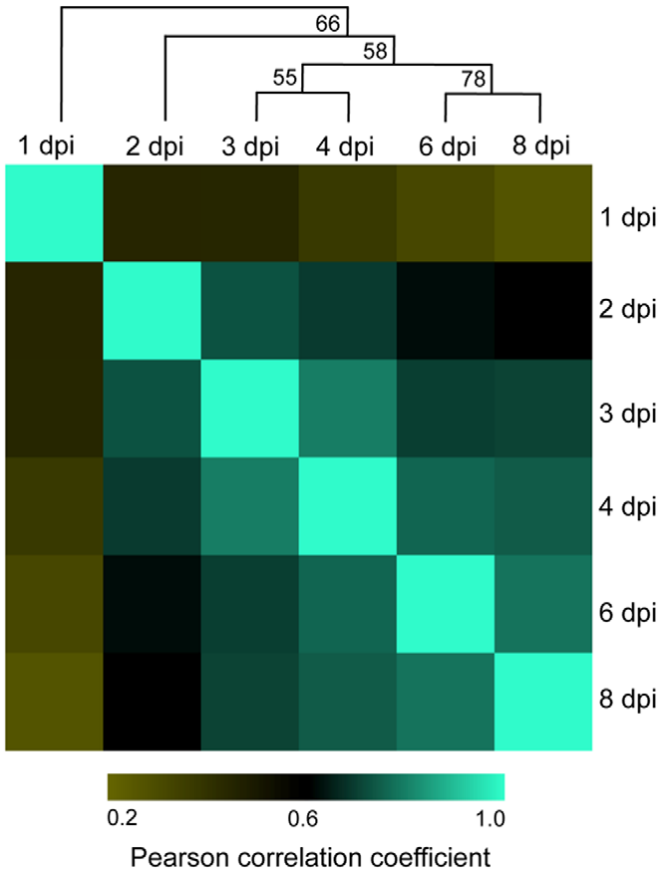
Correlation matrix of *Pseudoperonospora cubensis* expression profiles throughout a time course of *Cucumis sativus* infection. Normalized transcript abundances for 7,821 genes were calculated in fragments per kilobase pair of exon model per million fragments mapped (FPKM) with Cufflinks version 0.9.3 [56]. Pearson product-moment correlations (PCC) of log2 FPKM values were calculated for all pair-wise combinations using R (http://cran.r-project.org/). PCCs were clustered using hierarchical clustering with a Pearson correlation distance metric and average linkage using Multiple Experiment Viewer Software version 4.5 [57]. The bootstrap support values shown on tree nodes were obtained from 1000 bootstrap replicates. dpi = days post-inoculation.

In concordance with our visual assessment (Figure 2) of pathogen growth throughout the time course, our analyses showed a diversity of transcriptional changes in *Ps. cubensis*, as well as a correlation between gene expression levels and similar stages of pathogen growth. In support of this, we identified 7,821 genes expressed at different time points of infection (Table S1) and 129 of those genes (Table S2), mostly housekeeping, were expressed throughout all time points. Analyses of the top 20 highly expressed genes showed that genes expressed at earlier time points have substantially higher FPKM (fragments per kilobase pair of exon model per million fragments mapped) values than the genes expressed at later time points, consistent with the fewer numbers of genes expressed in the early stages of expression and saturation of detection of *Ps. cubensis* expression with our sampling depth (Table S3). For all time points analyzed, the minimum FPKM value was zero but the maximum FPKM values ranged from 8,528 at 8 dpi to 270,121 at 1 dpi (Table S3). The differences in transcriptome profiles are clearly visible in correlation and cluster analyses between the sampled time points. The Pearson Correlation Coefficient (PCC) values for comparisons of different time points ranged from 0.26 (1 dpi vs. 8 dpi) to 0.79 (3 dpi vs. 4 dpi) (Figure 4). Corresponding with our visual assessment of pathogen infection stages showing similar growth at 2 and 3 dpi (i.e., penetration and initial hyphal growth, Figure 2), gene expression patterns at 2 dpi are strongly associated with that of 3 dpi. Similarly, genes expressed at 6 dpi showed high correlation with the genes from 8 dpi, which represent comparable stages of pathogen growth and proliferation in the mesophyll (Figure 2). Additionally, at 1 dpi when encystment of zoospores is occurring, we observed a poor correlation (PCC ranged from 0.26 to 0.45) between expression at that time point with any other time point, likely due to the unique set of genes that would be involved in this process. Interestingly, gene expression at 3 dpi was highly correlated with other time points (PCC ranged from 0.45 to 0.79), suggesting that events occurring at 3 dpi may represent a transition phase between early and late infection.

### Differential gene expression

To identify genes specifically involved in distinct stages of pathogen infection and development, and to assess gene expression pattern changes over the course of infection, we next evaluated differentially expressed genes between all time points. To provide context for the differential expression, we included expression data from mRNA-Seq analysis of sporangia in which 8,254 *Ps. cubensis* genes were expressed (Table S4). In concordance with the similarities observed during our visualization of pathogen growth, comparisons with the least number of differentially expressed genes are those between early time points, with only 147 (2%) genes differentially expressed between 1 and 2 dpi (Table 1). Additionally, 1 and 2 dpi had fewer differentially expressed genes compared to sporangia than the later time points. Of all the combinations tested, 1 dpi had the highest percentage of differentially expressed genes across all pair-wise comparisons, despite having the lowest number of genes expressed, indicating that the events occurring at this stage of infection are unique among the time course. This corresponds both with our cluster analysis above (Figure 4) and our microscopic analysis of infection (Figure 2). Interestingly, despite the high correlation between expression patterns at 2 and 3 dpi seen using cluster analysis, there were a large number of genes differentially expressed between 2 and 3 dpi. This is additionally supportive of our hypothesis that 3 dpi is a transition phase between early and late infection. Not surprisingly, the highest number of differentially expressed genes were observed in comparison of all other time points and sporangia with 8 dpi, suggestive of an advanced stage of the infection process and a likely transition to processes involved in sporulation.

**Table 1:** Number of differentially expressed genes between each combination of time points and sporangia.

	2 dpi†	3 dpi	4 dpi	6 dpi	8 dpi	Sporangia
1 dpi	147 (50%)§	193 (58%)	189 (57%)	175 (60%)	192 (59%)	177 (50%)
2 dpi		848 (28%)	329 (10%)	306 (7%)	898 (19%)	246 (16%)
3 dpi			560 (14%)	301 (7%)	891 (17%)	391 (13%)
4 dpi				342 (7%)	820 (16%)	425 (14%)
6 dpi					644 (10%)	559 (15%)
8 dpi						1,301 (32%)

† dpi = days post-inoculation.

§ Numbers in parenthesis indicate the percent of significantly different tests out of the total number of tests that could be performed for each pairwise comparison.

Differential expression analysis was conducted using the CuffDiff program in Cufflinks version 0.9.3 [56] using the *Pseudoperonospora cubensis* annotation with a false discovery rate of 0.01.

### Expression of genes involved in virulence and pathogenicity

**Figure 5:**
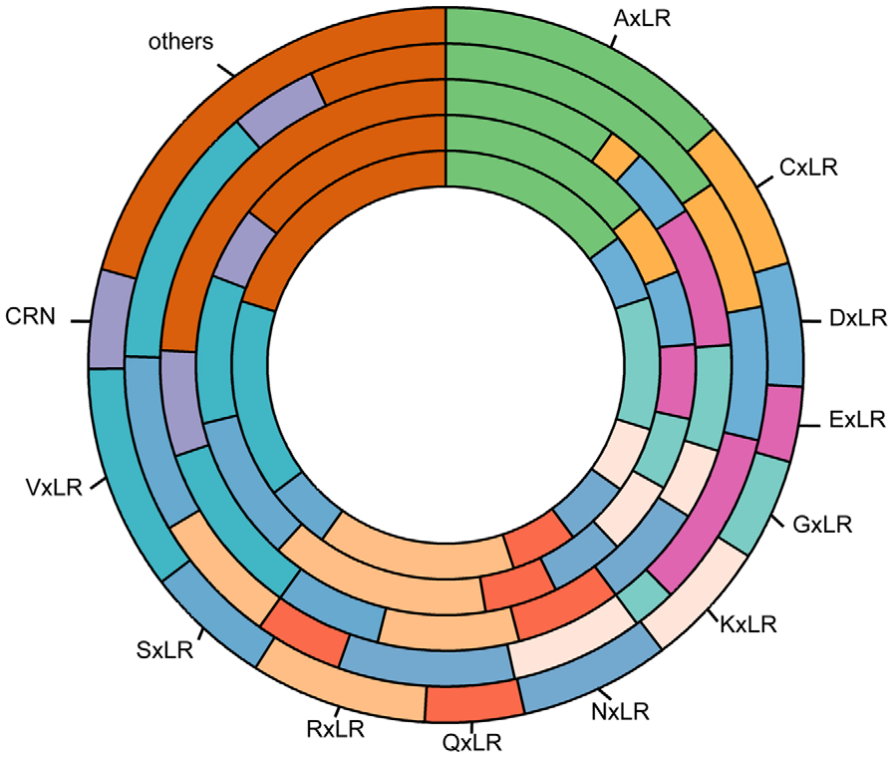
Candidate effectors expressed at different time points. From inner to outermost circles; 2 dpi, 3 dpi, 4 dpi, 6 dpi and 8 dpi. CRN = Crinkler effectors. dpi = days post-inoculation.

As an obligate biotroph, *Ps. cubensis* must evade and/or overcome basal plant defense responses, as well as effector-triggered immunity, in order to establish growth, proliferate and reproduce within its host. This, as in other phytopathogens, is likely achieved through the secretion of specific effector proteins that function within the host apoplast to interfere with extracellular plant defense responses, such as the activity of glucanases and proteases or cytoplasmically to suppress defense responses. Thus, the identification and characterization of the temporal expression of pathogen-associated genes throughout the course of infection can assist in the identification of secreted effectors that allow for both the promotion of disease, as well as the avoidance of host recognition. In support of this, we identified a suite of 271 candidate RXLR-type effectors within the *Ps. cubensis* genome [8] with 20 possible amino acid substitutions at position R1, including R and Q. In the current study, we analyzed the expression distribution of all 271-candidate effectors, as well as predicted Crinkler (CRN) effectors, over our time course of infection. As shown in Figure 5, the greatest number of expressed candidate effectors have an ASLR (Ala-Ser-Leu-Arg) motif, and are expressed at 2–8 dpi; candidate effectors with RXLR or QXLR motifs were expressed at every time point. Finally, as noted above, the expanded repertoire at the conserved RXLR motif in *Ps. cubensis*, with a total of 20 amino acids represented at the R1 position, represents substantial diversity in RXLR-type effectors, previously unreported. The simplest explanation for this expansion at R1 is supported by the hypothesis that RXLR-type effectors may play a role in host range, and that an expanded effector repertoire may impart plasticity. Moving forward, an extensive functional characterization of these RXLR-type effectors will provide insight into both pathogen virulence and host range specificity. Nonetheless, our data suggest that *Ps. cubensis* possesses a potentially highly expanded virulence capacity, of which, we have determined the expression of 271 RXLR-type effectors over an extensive time-course of susceptibility and disease elicitation in cucumber.

**Figure 6:**
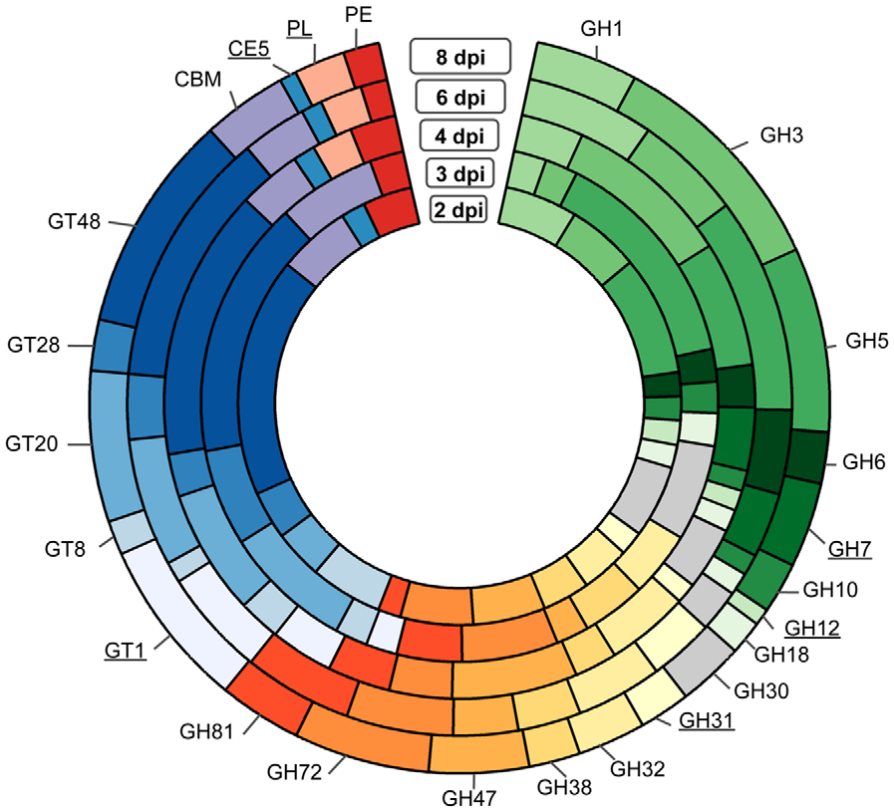
CAZymes in *Pseudoperonospora cubensis* expressed during infection on *Cucumis sativus*. The CAZymes coding genes in the *Ps. cubensis* genome were annotated using CAZymes Analysis Toolkit- CAT [66] according to the CAZy database [67] in combination with protein family domain analyses. Gene families absent in at least one time point are underlined. CBM = carbohydrate binding module. CE = carbohydrate esterase. GH = glycoside hydrolase. GT = glycosyl transferase. PE = pectin esterase. PL = polysaccharide lyase. dpi = days post-inoculation.

Gene families encoding host-targeted hydrolytic enzymes acting on plant proteinases, lipases, and several sugar-cleaving enzymes (carbohydrate active enzymes; CAZymes) were highly expressed in *Ps. cubensis* at 4 to 8 dpi, suggesting a possible role during infection and proliferation (Figure 6). Comparison of glycoside hydrolase (GH), glycosyltransferases (GT), polysaccharide lyase (PL), pectin esterase (PE), and carbohydrate esterase (CE) encoding genes revealed significant differences in number that were expressed as well as diversity across different time points. In total, 178 GH, 135 GT, 2 CE, and 15 PE were expressed throughout all the time points sampled (Figure 6). GH was the most represented family, with expression of 30–78 members followed by GT (17–27 members expressed). The most represented GH families identified were GH3 and GH5, while GT20 and GT48 were the most represented among all GTs. Additionally, substantial differences were observed in the types of CAZymes expressed across different time points. For example, several members of GH (GH family 7, 12, and 31), GT (GT family 1), CE (CE family 5), and PL family were absent in early time points (i.e., 2 and 3 dpi), yet were expressed at 4 to 8 dpi, suggesting a possible role during the later stage of infection. GH family 12 endoglucanases as well as CE family 5 cutinases have been previously implicated as having a role in infection by *Phytophthora* spp. [38], [47].

### Comparison to genes induced during *P. infestans* infection of potato

**Figure 7:**
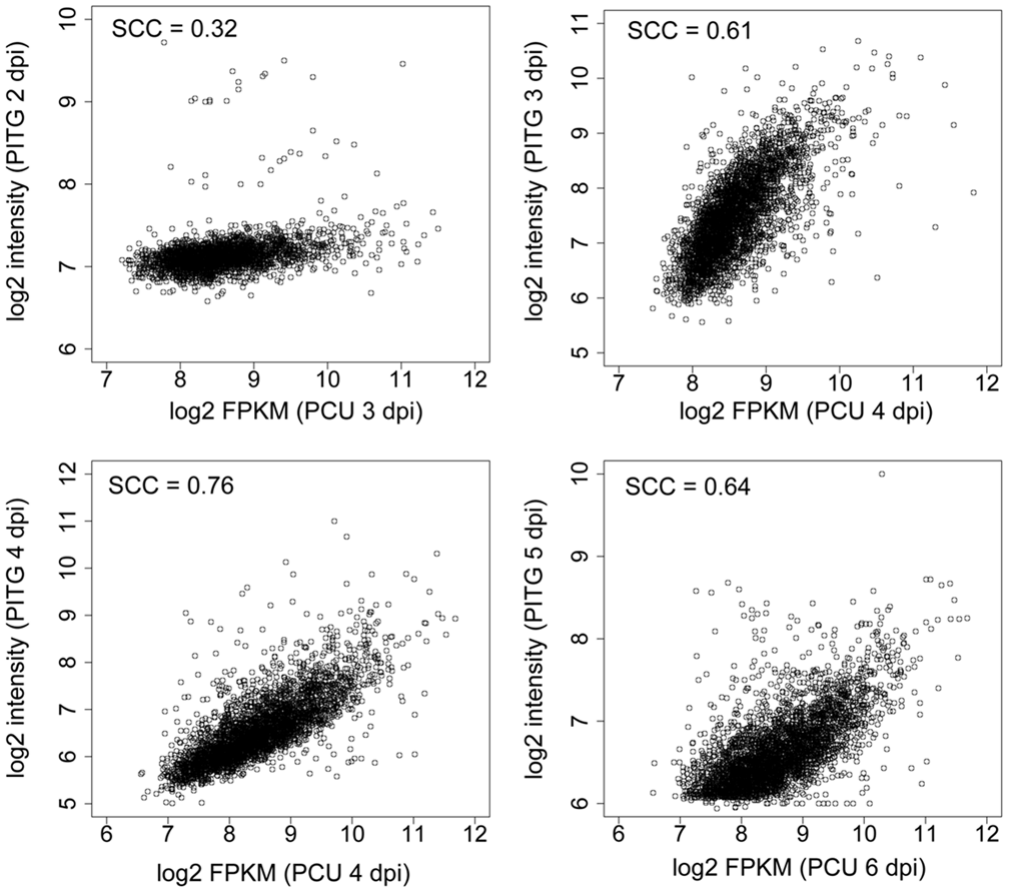
Comparison of ribonucleic acid sequencing (mRNA-Seq) and microarray expression patterns. Microarray expression profiles were obtained from time course analyses of genes expressed in *P. infestans* (PITG) during infection on *S. tuberosum* [10]. Single copy orthologous genes between *P. infestans* and *Ps. cubensis* (PCU) were identified using OrthoMCL [60], [61]. Log2 transformed expression values of single copy orthologous genes present in both the *Ps. cubensis* (log2 of fragments per kilobase pair of exon model per million fragments mapped [FPKM]) and *P. infestans* (log2 intensity) datasets are shown as scatter plots. SCC Spearman correlation coefficient. dpi = days post-inoculation.

The comparison of gene expression patterns between pathogens during infection of their susceptible hosts can allow for identification of common genes that are specifically involved in pathogenesis, as well as enable the discovery of genes unique to either species. To this end, we chose to compare the gene expression pattern of *Ps. cubensis* during infection to that of another economically important oomycete pathogen, *P. infestans*, during the infection of potato, *Solanum tuberosum*. Using clustering analysis of protein coding genes from both pathogens, we identified 7,374 single copy orthologous genes between these two oomycetes. We then compared the gene expression values obtained from our study (PCU) with those from microarray-based expression profiling [10] experiments with *P. infestans-S. tuberosum* (PITG). Spearman rank correlation coefficients (SCCs) of log2 expression values were calculated between the single copy orthologs at all time points in the two datasets; between 1,576 and 5,581 genes were included in the pair-wise comparisons (Figure 7). The SCC values among all comparisons ranged from 0.12 to 0.76 (Table S5). Comparisons between time points reflecting similar stages of pathogen infection showed higher overall correlations (0.29 to 0.76) as compared to comparisons between dissimilar time points (0.12 to 0.47). The most highly correlated comparisons were those between genes expressed in *Ps. cubensis* at 4 dpi and *P. infestans* at 4 dpi (SCC = 0.76). In *P. infestans* infection on potato, days 2–4 correspond to haustoria formation [10]; likewise, extensive formation of haustoria by *Ps. cubensis* was observed at 4 dpi (Figure 2). Correspondingly, genes expressed at 4 dpi include a haustorium-specific membrane protein, secreted RXLR proteins, as well as an amino acid transporter, which could possibly be involved in nutrient uptake *via* the haustorium to *Ps. cubensis*. Gene expression was also highly correlated (SCC = 0.64) between *Ps. cubensis* 6 dpi samples and *P. infestans* 5 dpi samples. At 6 dpi, symptoms of *Ps. cubensis* infection on cucumber manifest as chlorotic yellow lesions (Figure 2). Similarly, at 5 dpi, *Ph. infestans* has entered the mycelial necrotrophic growth stage, showing a similar chlorotic phenotype on its host [10].

### Gene co-expression network analyses

**Figure 8:**
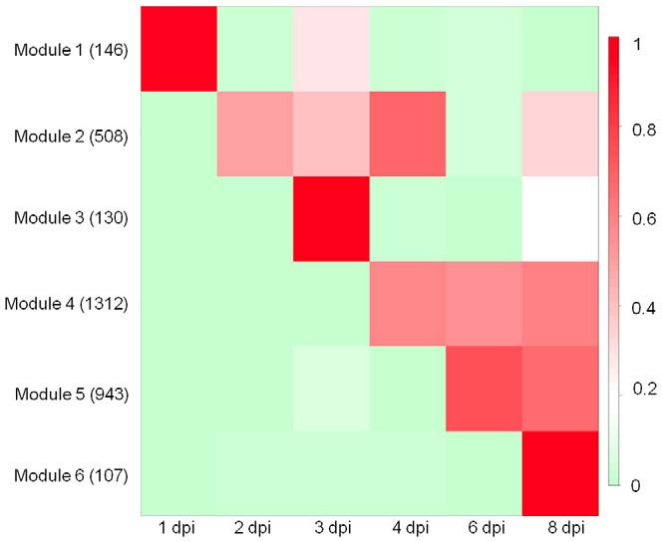
Heat map of the eigengenes representing each gene module. The columns in the heat map represent time points, and the rows represent eigengenes for each of the six identified co-expression modules [50]. The numbers of genes in each module are given in parentheses. The cells in the heat map show eigengene values between 0 and 1, indicators of relative expression levels of all genes in the module (see Materials and Methods). dpi = days post-inoculation.

Correlation analyses in which associations between gene expression patterns are identified are valuable for inferring common function and/or regulatory relationships [48]. In this study, we were primarily interested in identifying genes that are involved in both establishment and maintenance of *Ps. cubensis* infection, as well as those specifically involved in virulence. To this end, we constructed gene modules to identify highly co-expressed genes, where all members of a module are more highly correlated with each other than to genes outside the module. Using a Coefficient of Variation (CV) cutoff of 1.0, 4,195 genes from an initial total of 7,821 expressed genes were retained for downstream analyses. Using Weighted Gene Correlation Network Analysis (WGCNA) [49], 3,146 genes were assigned to six different gene modules (Modules 1 to 6) containing 107 to 1,312 genes; 1,049 genes were not assigned to any module (Table S6, Figure S3). Eigengenes [50] were calculated for each module and displayed in a heat map (Figure 8) revealing discrete gene expression patterns across different time points.

As described above, the 1 dpi sample represents both an important initial stage in the infection process, as well as a unique gene expression profile among the infection time points analyzed. This is additionally reflected in Module 1, which contains 146 genes that are highly expressed at 1 dpi, including genes involved in pathogenesis and transport (Figure 8, Figure S3, Table S6). The genes in this module are also expressed at 3 dpi, indicating that there may be some similarities between processes involved in zoospore adhesion and encystment, and initiation of haustoria formation. Genes expressed in Module 2 could represent those processes involved in the transition from early to late stages of infection and that are involved in the initial suppression of host defenses and establishment. This module, which contains 508 genes, expressed at 2, 3, and 4 dpi, represents gene expression occurring during initial penetration through the stomata into the host tissue, hyphal growth, and initiation of haustoria formation. It includes genes such as candidate RXLR-type effectors, glucanase inhibitors, CRNs, and a haustorium-specific membrane protein (Table S6), similar to what has been observed to be up-regulated during *P. infestans* infection on potato [10]. Our WGCNA analyses additionally identified genes that are co-expressed during the later stages of infection, specifically in Modules 4, 5, and 6 (Figure 3, Figure S2). Module 6, in particular, represents genes most highly expressed at 8 dpi possibly indicative of those involved in a shift to the reproductive phase and sporulation.

Transcription factors (TFs) are reported to play a key role in the regulation of many biological processes, including roles in oomycete pathogenesis [51], [52]; within the predicted *Ps. cubensis* proteome, 27 transcription factor-related domains in 440 genes were identified (Table S7, S8). A total of 247 of these were expressed throughout the infection process (Table S7). We also identified genes encoding transcription factor-related Pfam domains in all six co-expression modules. Two modules, 2 and 4, with genes co-expressed across different time contained the majority of the transcription factors. The transcription factor-related genes within those modules could play important role in regulation of genes involved in pathogenesis. The bZIP and Myb, DNA-binding transcription factor, which play an important role in oomycete pathogenesis [53], [54], were the most abundant transcription factor-related domains expressed during infection.

### Conclusions

In this study, we present an extensive characterization of the gene expression analysis of the obligate oomycete cucurbit pathogen *Ps. cubensis* during a compatible interaction. This data set represents the first global gene expression profile of a cucurbit pathogen. Using mRNA-Seq, we analyzed the differential expression of pathogen genes across a time course of infection of cucumber, correlating expression with pathogen infection structures, development, and the onset of disease symptoms. Our study provides a comprehensive examination of the key infection stages of *Ps. cubensis* growth and development and through clustering and co-expression network analyses, describes genes that are specifically expressed during these stages. In addition, our work has identified an expanded effector repertoire, represented by a unique diversity at the canonical RXLR motif. Overall, the work described herein will significantly enhance our understanding of the regulation of infection of oomycete phytopathogens, as well as a baseline for identifying important virulence determinants in *Ps. cubensis*.

## Materials and Methods

### *Ps. cubensis* inoculation and sample collection

*Ps. cubensis* MSU-1 was maintained on *Cucumis sativus* cultivar ‘Vlaspik’ as described previously [9]. Four-week-old cucumber plants were inoculated on the abaxial surface of the first fully-expanded leaf with a 1×10^5^ sporangia/ml solution with 20–30 10 µl droplets. Inoculated plants were maintained at 100% relative humidity in the dark for 24 hours and then transferred to growth chambers maintained at 22°C with a 12 h light/dark photoperiod. Samples were collected at 1, 2, 3, 4, 6, and 8 dpi with a #3 cork borer to collect tissue at the site of inoculation. Samples for RNA extraction were frozen in liquid nitrogen and stored at −80°C until use. Samples collected for microscopy were cleared in 95% ethanol and stored at room temperature.

### Histological assessment of *Ps. cubensis* growth

Cleared infected leaf discs were stained in a solution of 250 µg/ml trypan blue in equal parts lactic acid, water, and glycerol to visualize infection structures. Microscopy was performed using an Olympus IX71 inverted light microscope. Images were captured using an Olympus DC71 camera and were processed for contrast using Canvas X (ACD Systems International, Inc., Seattle, WA).

### Library preparation and sequencing

Sporangia were washed from the abaxial surface of heavily sporulating leaves, filtered through a 40 µm nylon cell strainer, and pelleted *via* centrifugation. For RNA extraction (RNeasy Mini Kit, Qiagen, Valencia, CA), sporangia were resuspended in 450 µl RLT buffer with ∼50 µl 425–600 µm acid-washed beads and vortexed for 3 minutes to break cells. Additional extraction steps were followed according to the manufacturer's instructions. RNA concentration and quality was determined using the Bioanalyzer 2100 (Agilent Technologies, San Diego CA). The sporangia library was sequenced in two lanes at the UC DNA Sequencing Facility at University of California, Davis (Davis, CA). RNA samples from the infection time course were processed as described in Adhikari et al. [46]. In brief, RNA was isolated using the RNeasy Mini Kit (Qiagen, Germantown, MD), treated with DNase (Promega, Madison, WI) and barcoded libraries constructed with the Illumina mRNA-seq kit (Illumina, San Diego CA). Libraries were sequenced with the Illumina Genome Analyzer II platform generating 35–42 bp single-end reads. Reads from biological replicates were pooled prior to expression abundance measurements. Reads were deposited in the National Center for Biotechnology Information Sequence Read Archive under accession number SRP009350.

### mRNA-Seq read mapping and transcript abundance estimation

The assembled and annotated *Ps. cubensis* MSU-1 genome sequence ([8]; http://www.daylab.plp.msu.edu/wp-content/uploads/psc_merged_contigs.fasta.zip, http://www.daylab.plp.msu.edu/wp-content/uploads/psc_merged_contigs.gff3.zip) was used to estimate transcript abundances. mRNA-Seq reads for each time point and control (sporangia) were mapped to the 67.9 Mb *Ps. cubensis* reference genome [8] using the quality aware alignment algorithms, Bowtie version 0.12.7 [55] and TopHat version 1.2.0 [55]. The single-end reads from different time points were aligned in single-end mode while the paired-end reads from the control were aligned in paired-end mode. The minimum and maximum intron length was set to 5 and 50,000 bp, respectively and the insert size for paired-end mode was set to 140 bp.

The aligned read files produced by TopHat were processed by Cufflinks v0.9.3 [56]. A reference annotation of the *Ps. cubensis* genome (23,519 loci and 23,522 gene models; [8]) was provided and the maximum intron length was set to 50,000 bp. Normalized gene expression levels were calculated and reported as FPKM. The quartile normalization option was used to improve differential expression calculations of lowly expressed genes [56]; all other parameters were used at the default settings. A gene was considered expressed in a specific sample if the FPKM value and FPKM 95% confidence interval lower boundary was greater than 0.001 and zero, respectively.

Pearson product-moment correlation analyses of log2 FPKM values among mRNA-Seq libraries were performed using R (http://cran.r-project.org/), with all log2 FPKM values less than zero set to zero. Only tests significant at *p* = 0.05 are shown. Correlation values depicted as a heat map were clustered with hierarchical clustering using a Pearson correlation distance metric and average linkage. The bootstrap support values were calculated from 1000 replicates using Multiple Experiment Viewer Software (MeV) v4.5 [57]. To understand variability among biological replicates, Pearson correlation coefficients were calculated for the log2 transformed FPKM values of the genes expressed in both replicates at a particular time point.

### Identification of differentially expressed genes

Once transcript abundance estimation was calculated, differential expression analysis was conducted using the Cuffdiff program within Cufflinks version 0.9.3 [56] utilizing the read alignment files described above. The expression testing was done at the level of genes. Quartile normalization [58] and a false discovery rate of 0.01 after Benjamini-Hochberg correction for multiple testing were used. The *Ps. cubensis* genome and the annotation files were provided as input parameters. All other parameters were used at the default levels. Cuffdiff was used to perform pairwise comparisons of six time points and sporangia.

### mRNA-Seq and microarray comparative analyses

Gene expression data from a *P. infestans*-*S. tuberosum* time course experiment [10] was used to assess gene expression pattern similarities/differences in two ooymcete pathogens. The data set included *P. infestans* gene expression over a five-day (2–5 days) time course of a potato infection. Raw data was downloaded from the Gene Expression Omnibus (http://www.ncbi.nlm.nih.gov/geo/) (GSE14480) [59]. The probe intensities were normalized using Robust Multichip Analyses method [60]. For the mRNA-Seq to microarray comparative analysis, single copy orthologous genes were identified using OrthoMCL [60], [61] with default parameters. Clustering of 23,522 and 18,140 protein-coding genes from *Ps. cubensis* and *P. infestans*, respectively, yielded 7,374 clusters with single copy genes from both species. Only single copy orthologous genes were used for the analyses. FPKM and probe intensity values were log2 transformed, and Spearman correlation coefficients were calculated using R (http://cran.r-project.org/).

### Functional Analysis

Functional annotation for all *Ps. cubensis* genes were generated from searches of the UniProt databases (Uuniref100) [62] with BLAST and combined with Pfam [63] protein families assignment performed using HMMER3 [64]. Functional annotations for *Ps. cubensis* sequences were taken from the best possible UniRef sequence match, but if there was no UniRef sequence match, functional annotations were made based on the best Pfam domain alignment. Transcription factors were identified based on PFAM domains.

### Gene co-expression network analysis

Gene co-expression network analysis was done according to the methods described by Childs et al. [65] with some modifications. First, the FPKM gene expression values were log2 transformed and FPKM values less than 1 were transformed to zero. Second, genes showing no variation across time points were filtered out using a coefficient of variance (CV) cutoff (1.0). Third, the β and treecut parameters were 7 and 0.6, respectively. Eigengenes were calculated using the WGCNA package [50]. The heat map of eigengenes for each gene module was constructed using R (http://cran.r-project.org/). Genes assigned to co-expression modules were annotated based on the *Ps. cubensis* functional annotation.

## Supporting Information

Figure S1 **Number of total mRNA-Seq reads, reads mapped, and number of genes expressed at different time points.** Total number of reads, number of reads mapped, and number of genes expressed in *Cucumis sativus* (Cusa) and *Pseudoperonospora cubensis* (Pcu) at different time-points are shown. Reads were mapped using Bowtie version 0.12.5 [55] and TopHat version 1.2.0 [55]. Fragments per kilobase pair of exon model per million fragments mapped (FPKM) values were calculated using Cufflinks version 0.9.3 [56]. Genes were considered expressed if the 95% confidence interval lower boundary FPKM value was greater than zero. dpi = days post-inoculation.
(TIF)

Figure S2 **Concordance of FPKM values of the genes expressed in two biological replicates of the** ***Pseudoperonospora cubensis*** **transcriptome.** Reads from different time-points were mapped to *Ps. cubensis* genome using Bowtie version 0.12.5 [55] and TopHat version 1.2.0 [55]. Fragments per kilobase pair of exon model per million fragments mapped (FPKM) values were calculated using Cufflinks version 0.9.3 [56] and *Ps. cubensis* genome annotations. For each time point Log2 transformed FPKM values of equal number of genes from both replicates are plotted. Pearson Correlation Coefficient (PCC) was calculated using R. dpi, days post-inoculation.
(PDF)

Figure S3 **Trend plots of the normalized gene expression values for each gene from six identified gene co-expression modules.** Modules consisting of genes expressed modules 1, 2, 3, 4, 5, and 6 are shown.
(PDF)

Table S1 **List of genes expressed in at least one time point of infection or in isolated sporangia.** Shown are the gene ID, FPKM values of those genes at different time points and functional annotation.
(XLSX)

Table S2 **List of genes expressed across all time points.** Gene expression values (FPKM) were calculated by using Cufflinks [55].
(XLSX)

Table S3 **List of 20 highly expressed genes at different time points and control samples.** Their FPKM values and putative function as determined by BLASTX searches against UniRef100 (E-value cutoff 1e-5) are shown.
(XLSX)

Table S4 **List of genes expressed in sporangia.** Shown are the gene ID, FPKM values along with their functional annotation.
(XLSX)

Table S5 **Spearman rank correlations of log2 expression values between ribonucleic acid sequencing (mRNA-Seq) and microarray based expression profiles.**
(XLSX)

Table S6 **List of modules (module 1 to 6) with their corresponding gene ID, putative function as determined by BLASTX searches against UniRef100 (E-value cutoff 1e-5) and transcription factor-related Pfam domains.**
(XLSX)

Table S7 **Number of transcription factor-related domains present in the genes expressed at different time points of** ***Cucumis sativus*** **infection by** ***Pseudoperonospora cubensis*** **.** Shown is the Pfam domain accession, Pfam domain name and description.
(XLSX)

Table S8 **List of genes containing transcription factor-related Pfam domains with their corresponding gene IDs, Pfam domain accession and domain names.** Transcription factor-related Pfam domains were identified based on Pfam domain assignment.
(XLS)

## References

[1] Savory EA, Granke LL, Quesada-Ocampo LM, Varbanova M, Hausbeck MK (2011). The cucurbit downy mildew pathogen *Pseudoperonospora cubensis*.. Mol Plant Pathol.

[2] Lebeda A, Cohen Y (2011). Cucurbit downy mildew (*Pseudoperonospora cubensis*)—biology, ecology, epidemiology, host-pathogen interaction and control.. Eur J Plant Pathol.

[3] Thomas C, Inaba T, Cohen Y (1987). Physiological specialization in *Pseudoperonospora cubensis*.. Phytopathol.

[4] Sarris P, Abdelhalim M, Kitner M, Skandalis N, Panopoulos N (2009). Molecular polymorphisms between populations of *Pseudoperonospora cubensis* from Greece and the Czech Republic and the phytopathological and phylogenetic implications.. Plant Pathol.

[5] Lebeda A, Widrlechner MP (2003). A set of Cucurbitaceae taxa for differentiation of *Pseudoperonespora cubensis* pathotypes.. J Plant Dis Prot.

[6] Runge F, Choi Y-J, Thines M (2011). Phylogenetic investigations in the genus *Pseudoperonospora* reveal overlooked species and cryptic diversity in the *P. cubensis* species cluster.. Eur J Plant Pathol.

[7] Choi Y (2005). A re-consideration of *Pseudoperonospora cubensis* and *P. humuli* based on molecular and morphological data.. Mycological Res.

[8] Savory EA, Zou C, Adhikari BN, Hamilton JP, Buell CR (2012). Alternative splicing of a multi-drug transporter from *Pseudoperonospora cubensis* generates an RXLR effector protein that elicits a rapid cell death.. PLoS ONE.

[9] Tian M, Win J, Savory E, Burkhardt A, Held M (2011). 454 Genome sequencing of *Pseudoperonospora cubensis* reveals effector proteins with a QXLR translocation motif.. Mol Plant Microbe Interact.

[10] Haas BJ, Kamoun S, Zody MC, Jiang RH, Handsaker RE (2009). Genome sequence and analysis of the Irish potato famine pathogen *Phytophthora infestans*.. Nature.

[11] Kamoun S (2006). A catalogue of the effector secretome of plant pathogenic oomycetes.. Ann Rev Phytopathol.

[12] Cabral A, Stassen JH, Seidl MF, Bautor J, Parker JE (2011). Identification of *Hyaloperonospora arabidopsidis* transcript sequences expressed during Infection reveals isolate-specific effectors.. PLoS ONE.

[13] Sierra R, Rodriguez RL, Chaves D, Pinzon A, Grajales A (2010). Discovery of *Phytophthora infestans* genes expressed in planta through mining of cDNA libraries.. PLoS ONE.

[14] Torto-Alalibo TA, Tripathy S, Smith BM, Arredondo FD, Zhou L (2007). Expressed sequence tags from *Phytophthora sojae* reveal genes specific to development and infection.. Mol Plant Microbe Interact.

[15] Randall TA, Dwyer RA, Huitema E, Beyer K, Cvitanich C (2005). Large-scale gene discovery in the oomycete *Phytophthora infestans* reveals likely components of phytopathogenicity shared with true fungi.. Mol Plant Microbe Interact.

[16] Bos JI, Kanneganti TD, Young C, Cakir C, Huitema E (2006). The C-terminal half of *Phytophthora infestans* RXLR effector AVR3a is sufficient to trigger R3a-mediated hypersensitivity and suppress INF1-induced cell death in *Nicotiana benthamiana*.. Plant J.

[17] Fabro G, Steinbrenner J, Coates M, Ishaque N, Baxter L (2011). Multiple Candidate Effectors from the Oomycete Pathogen *Hyaloperonospora arabidopsidis* Suppress Host Plant Immunity.. PLoS Pathog.

[18] Armstrong MR, Whisson SC, Pritchard L, Bos JI, Venter E (2005). An ancestral oomycete locus contains late blight avirulence gene Avr3a, encoding a protein that is recognized in the host cytoplasm.. Proc Natl Acad Sci U S A.

[19] Allen RL (2004). Host-Parasite Coevolutionary Conflict Between Arabidopsis and Downy Mildew.. Science.

[20] Money NP, Davis CM, Ravishankar JP (2004). Biomechanical evidence for convergent evolution of the invasive growth process among fungi and oomycete water molds.. Fung Gen Biol.

[21] Dong S, Qutob D, Tedman-Jones J, Kuflu K, Wang Y (2009). The *Phytophthora sojae* avirulence locus Avr3c encodes a multi-copy RXLR effector with sequence polymorphisms among pathogen strains.. PLoS ONE.

[22] Torto TA (2003). EST mining and functional expression fssays identify extracellular effector proteins from the plant pathogen *Phytophthora*.. Genome Res.

[23] Schornack S, van Damme M, Bozkurt TO, Cano LM, Smoker M (2010). Ancient class of translocated oomycete effectors targets the host nucleus.. Proc Natl Acad Sci U S A.

[24] Damasceno CM, Bishop JG, Ripoll DR, Win J, Kamoun S (2008). Structure of the glucanase inhibitor protein (GIP) family from phytophthora species suggests coevolution with plant endo-beta-1,3-glucanases.. Mol Plant Microbe Interact.

[25] Rose JK, Ham KS, Darvill AG, Albersheim P (2002). Molecular cloning and characterization of glucanase inhibitor proteins: coevolution of a counterdefense mechanism by plant pathogens.. Plant Cell.

[26] Tian M, Benedetti B, Kamoun S (2005). A Second Kazal-like protease inhibitor from *Phytophthora infestans* inhibits and interacts with the apoplastic pathogenesis-related protease P69B of tomato.. Plant Physiol.

[27] Tian M, Huitema E, Da Cunha L, Torto-Alalibo T, Kamoun S (2004). A Kazal-like extracellular serine protease inhibitor from *Phytophthora infestans* targets the tomato pathogenesis-related protease P69B.. J Biol Chem.

[28] Tian M, Win J, Song J, van der Hoorn R, van der Knaap E (2007). A *Phytophthora infestans* cystatin-like protein targets a novel tomato papain-like apoplastic protease.. Plant Physiol.

[29] Liu Z, Bos JI, Armstrong M, Whisson SC, da Cunha L (2005). Patterns of diversifying selection in the phytotoxin-like scr74 gene family of *Phytophthora infestans*.. Mol Biol Evol.

[30] Levesque CA, Brouwer H, Cano L, Hamilton JP, Holt C (2010). Genome sequence of the necrotrophic plant pathogen *Pythium ultimum* reveals original pathogenicity mechanisms and effector repertoire.. Genome Biol.

[31] Fellbrich G, Romanski A, Varet A, Blume B, Brunner F (2002). NPP1, a Phytophthora-associated trigger of plant defense in parsley and Arabidopsis.. Plant J.

[32] Cabral A, Oome S, Sander N, Kuefner I, Nürnberger T (2012). Non-toxic Nep1-like proteins of the downy mildew pathogen *Hyaloperonospora arabidopsidis*; repression of necrosis-inducing activity by a surface-exposed region.. Mol Plant Microbe Interact.

[33] Gaulin E, Drame N, Lafitte C, Torto-Alalibo T, Martinez Y (2006). Cellulose binding domains of a *Phytophthora* cell wall protein are novel pathogen-associated molecular patterns.. Plant Cell.

[34] Gaulin E, Jauneau A, Villalba F, Rickauer M, Esquerré-Tugayé MT (2002). The CBEL glycoprotein of *Phytophthora parasitica* var-nicotianae is involved in cell wall deposition and adhesion to cellulosic substrates.. J Cell Sci.

[35] Tyler BM (2006). *Phytophthora* genome sequences uncover evolutionary origins and mechanisms of pathogenesis.. Science.

[36] Baxter L, Tripathy S, Ishaque N, Boot N, Cabral A (2010). Signatures of adaptation to obligate biotrophy in the *Hyaloperonospora arabidopsidis* genome.. Science.

[37] Moy P, Qutob D, Chapman BP, Atkinson I, Gijzen M (2004). Patterns of gene expression upon infection of soybean plants by *Phytophthora sojae*.. Mol Plant Microbe Interact.

[38] Costanzo S, Ospina-Giraldo MD, Deahl KL, Baker CJ, Jones RW (2006). Gene duplication event in family 12 glycosyl hydrolase from *Phytophthora* spp.. Fung Gen Biol.

[39] Yamaguchi Y, Huffaker A (2011). Endogenous peptide elicitors in higher plants.. Current opinion in plant biology.

[40] Fernandez D, Tisserant E, Talhinhas P, Azinheira H, Vieira ANA (2011). 454-pyrosequencing of *Coffea arabica* leaves infected by the rust fungus *Hemileia vastatrix* reveals in planta-expressed pathogen-secreted proteins and plant functions in a late compatible plant–rust interaction.. Mol Plant Pathol.

[41] Joly DL, Feau N, Tanguay P, Hamelin RC (2010). Comparative analysis of secreted protein evolution using expressed sequence tags from four poplar leaf rusts (*Melampsora* spp.).. BMC Gen.

[42] Miranda M, Ralph SG, Mellway R, White R, Heath MC (2007). The transcriptional response of hybrid poplar (*Populus trichocarpa*×*P. deltoides*) to infection by *Melampsora medusae* leaf rust involves induction of flavonoid pathway genes leading to the accumulation of proanthocyanidins.. Mol Plant Microbe Interact.

[43] Duplessis S, Hacquard S, Delaruelle C, Tisserant E, Frey P (2011). *Melampsora larici*-populina tanscript profiling during germination and timecourse infection of poplar leaves reveals dynamic expression patterns associated with virulence and biotrophy.. Mol Plant Microbe Interact.

[44] Mosquera G, Giraldo M, Khang CH, Coughlan S, Valent B (2009). Interaction transcriptome analysis identifies *Magnaporthe oryzae* BAS1-4 as biotrophy-associated secreted proteins in rice blast disease.. Plant Cell.

[45] Polesani M, Desario F, Ferrarini A, Zamboni A, Pezzotti M (2008). cDNA-AFLP analysis of plant and pathogen genes expressed in grapevine infected with *Plasmopara viticola*.. BMC Gen.

[46] Adhikari B, Savory E, Vaillancourt B, Childs KL, Hamilton JP (2012). Expression profiling of Cucumis sativus in response to infection by Pseudoperonospora cubensis.. PLOS ONE.

[47] Ospina-Giraldo M, Griffith J, Laird E, Mingora C (2010). The CAZyome of *Phytophthora* spp.: A comprehensive analysis of the gene complement coding for carbohydrate-active enzymes in species of the genus *Phytophthora*.. BMC Gen.

[48] Ihmels J, Bergmann S, Berman J, Barkai N (2005). Comparative gene expression analysis by a differential clustering approach: Application to the *Candida albicans* transcription program.. PLoS Genet.

[49] Langfelder P, Zhang B, Horvath S (2008). Defining clusters from a hierarchical cluster tree: the Dynamic Tree Cut package for R.. Bioinformatics.

[50] Langfelder P, Horvath S (2007). Eigengene networks for studying the relationships between co-expression modules.. BMC Sys Biol.

[51] Iyer LM, Anantharaman V, Wolf MY, Aravind L (2008). Comparative genomics of transcription factors and chromatin proteins in parasitic protists and other eukaryotes.. In J Parasitol.

[52] Wang Y, Dou D, Wang X, Li A, Sheng Y (2009). The PsCZF1 gene encoding a C2H2 zinc finger protein is required for growth, development and pathogenesis in *Phytophthora sojae*.. Micro Path.

[53] Blanco FA, Judelson HS (2005). A bZIP transcription factor from *Phytophthora* interacts with a protein kinase and is required for zoospore motility and plant infection.. Mol Micro.

[54] Judelson H, Ah-Fong A (2010). The kinome of *Phytophthora infestans* reveals oomycete-specific innovations and links to other taxonomic groups.. BMC Gen.

[55] Langmead B, Trapnell C, Pop M, Salzberg SL (2009). Ultrafast and memory-efficient alignment of short DNA sequences to the human genome.. Genome Biol.

[56] Trapnell C, Williams BA, Pertea G, Mortazavi A, Kwan G (2010). Transcript assembly and quantification by RNA-Seq reveals unannotated transcripts and isoform switching during cell differentiation.. Nat Biotechnol.

[57] Saeed AI, Bhagabati NK, Braisted JC, Liang W, Sharov V (2006). TM4 microarray software suite.. Methods Enz.

[58] Bullard J, Purdom E, Hansen K, Dudoit S (2010). Evaluation of statistical methods for normalization and differential expression in mRNA-Seq experiments.. BMC Bioinformatics.

[59] Edgar R, Domrachev M, Lash AE (2002). Gene Expression Omnibus: NCBI gene expression and hybridization array data repository.. Nucleic Acids Res.

[60] Irizarry R, Hobbs B, Collin F, Beazer-Barclay Y, Antonellis K (2003). Exploration, normalization, and summaries of high density oligonucleotide array probe level data.. Biostatistics.

[61] Chen F, Mackey AJ, Vermunt JK, Roos DS (2007). Assessing performance of orthology detection strategies applied to eukaryotic genomes.. PLoS ONE.

[62] Suzek BE, Huang H, McGarvey P, Mazumder R, Wu CH (2007). UniRef: comprehensive and non-redundant UniProt reference clusters.. Bioinformatics.

[63] Bateman A, Birney E, Durbin R, Eddy SR, Howe KL (2000). The Pfam protein families database.. Nucleic Acids Res.

[64] Eddy SR (2009). A new generation of homology search tools based on probabilistic inference.. Genome Inform.

[65] Childs KL, Davidson RM, Buell CR (2011). Gene coexpression network analysis as a source of functional annotation for rice genes.. PLoS ONE.

[66] Park BH, Karpinets TV, Syed MH, Leuze MR, Uberbacher EC (2010). CAZymes Analysis Toolkit (CAT): Web-service for searching and analyzing carbohydrate-active enzymes in a newly sequenced organism using CAZy database.. Glycobiology.

[67] Cantarel BL, Coutinho PM, Rancurel C, Bernard T, Lombard V (2009). The Carbohydrate-Active EnZymes database (CAZy): an expert resource for glycogenomics.. Nucleic Acids Res.

