# Supplementary figures and images for: mRNA-Seq Analysis of the Pseudoperonospora cubensis Transcriptome During Cucumber (Cucumis sativus L.) Infection
Source: PLoS One. 2012 Apr 24;7(4):e35796. doi: 10.1371/journal.pone.0035796 (PMC3335787; doi:10.1371/journal.pone.0035796)

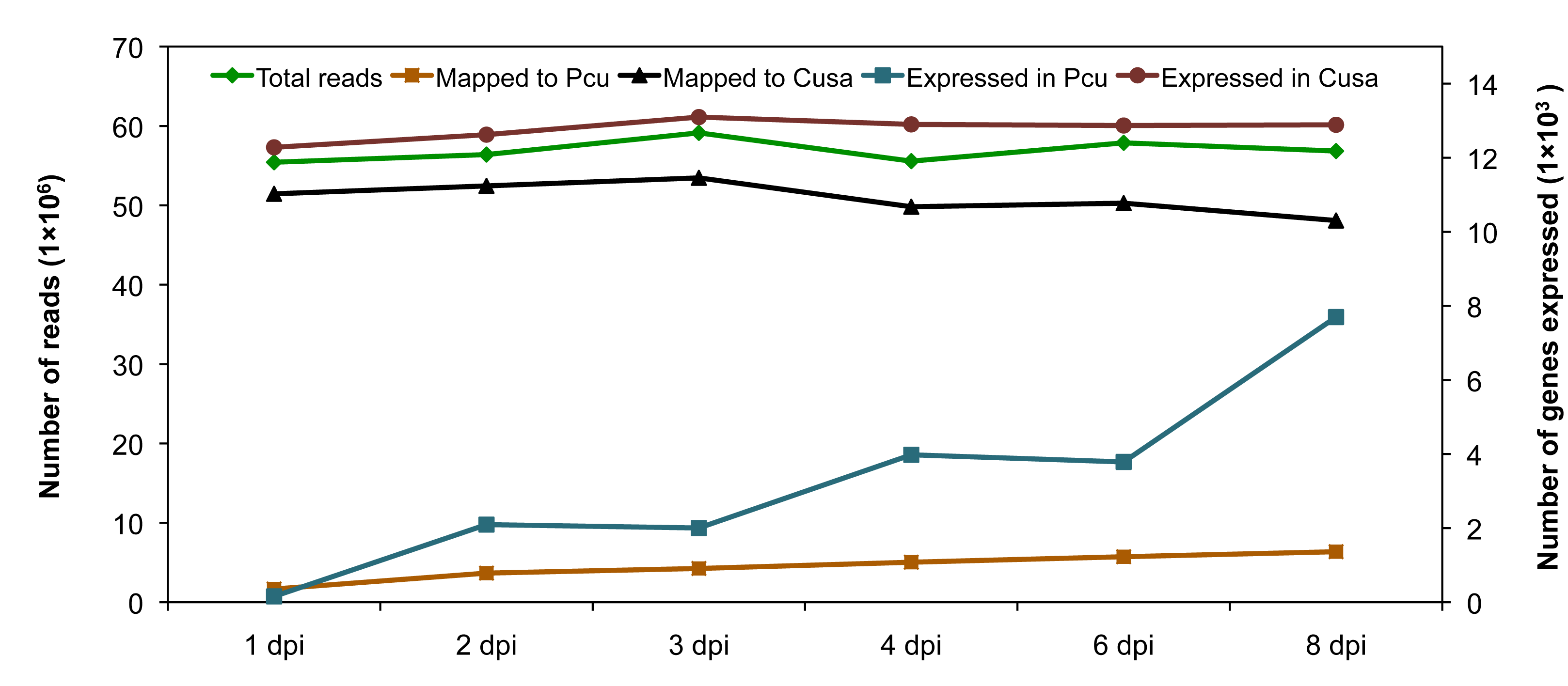

Supplement: Figure S1 — Number of total mRNA-Seq reads, reads mapped, and number of genes expressed at different time points. Total number of reads, number of reads mapped, and number of genes expressed in Cucumis sativus (Cusa) and Pseudoperonospora cubensis (Pcu) at different time-points are shown. Reads were mapped using Bowtie version 0.12.5 [55] and TopHat version 1.2.0 [55]. Fragments per kilobase pair of exon model per million fragments mapped (FPKM) values were calculated using Cufflinks version 0.9.3 [56]. Genes were considered expressed if the 95% confidence interval lower boundary FPKM value was greater than zero. dpi = days post-inoculation. (TIF) [file pone.0035796.s001.tif]

Biological replicate 2  $\log_2$  FPKM

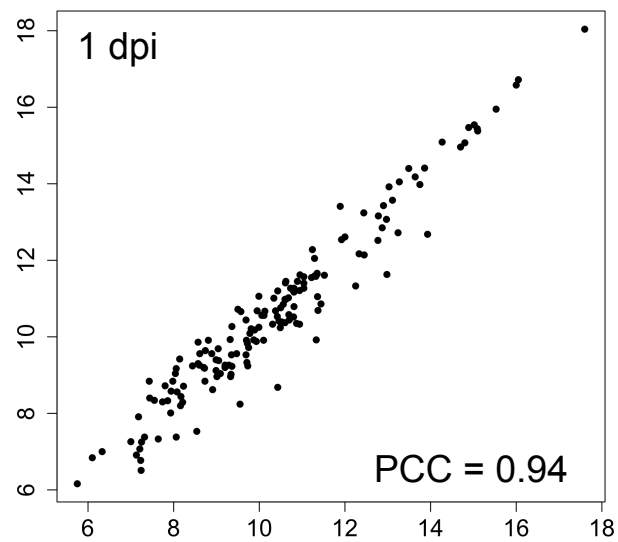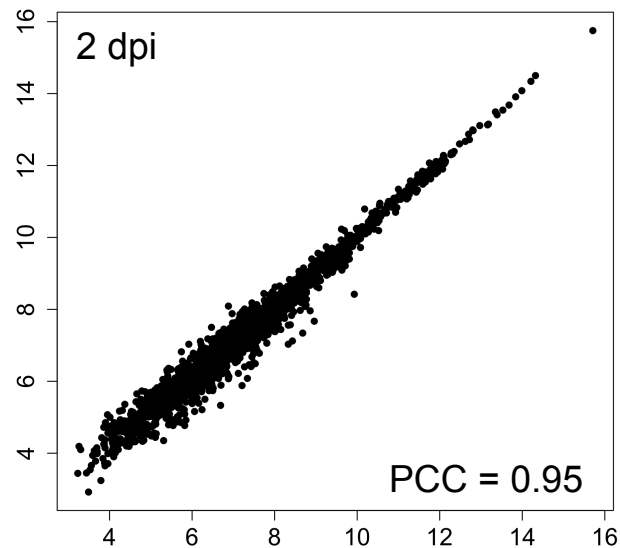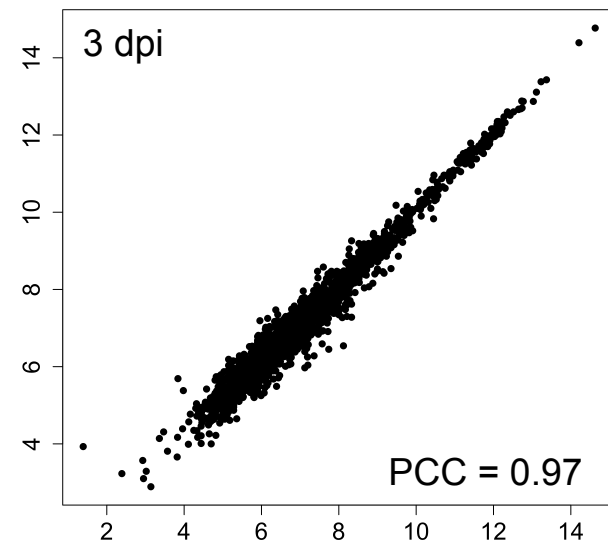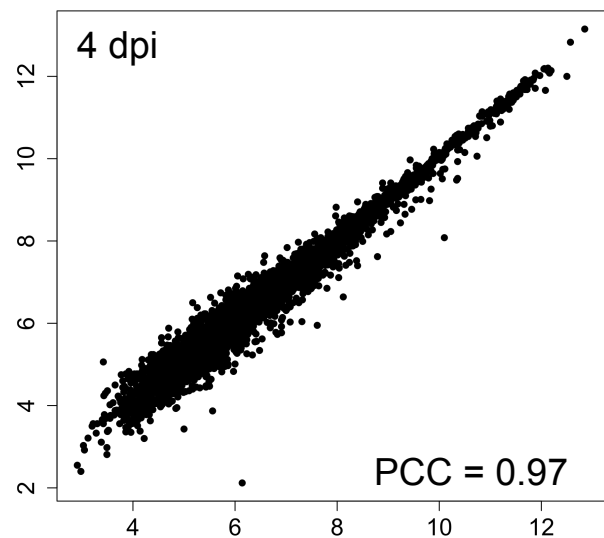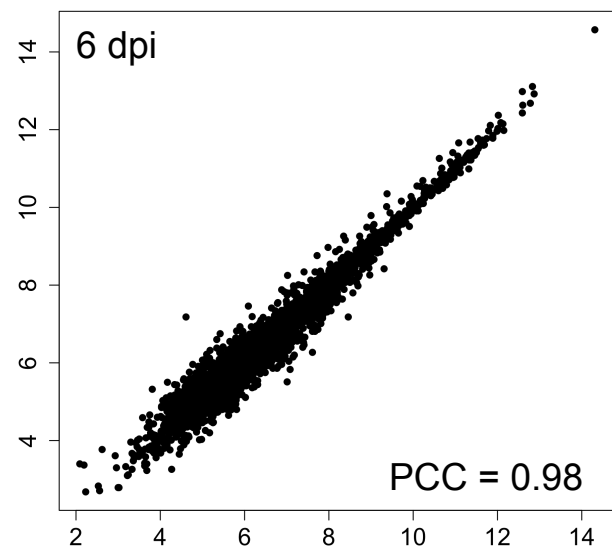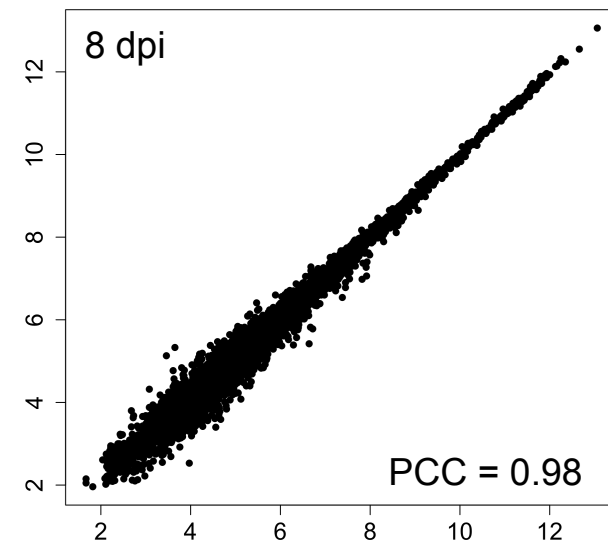

Biological replicate 1  $\log_2$  FPKM

Supplement: Figure S2 — Concordance of FPKM values of the genes expressed in two biological replicates of the Pseudoperonospora cubensis transcriptome. Reads from different time-points were mapped to Ps. cubensis genome using Bowtie version 0.12.5 [55] and TopHat version 1.2.0 [55]. Fragments per kilobase pair of exon model per million fragments mapped (FPKM) values were calculated using Cufflinks version 0.9.3 [56] and Ps. cubensis genome annotations. For each time point Log2 transformed FPKM values of equal number of genes from both replicates are plotted. Pearson Correlation Coefficient (PCC) was calculated using R. dpi, days post-inoculation. (PDF) [file pone.0035796.s002.pdf]

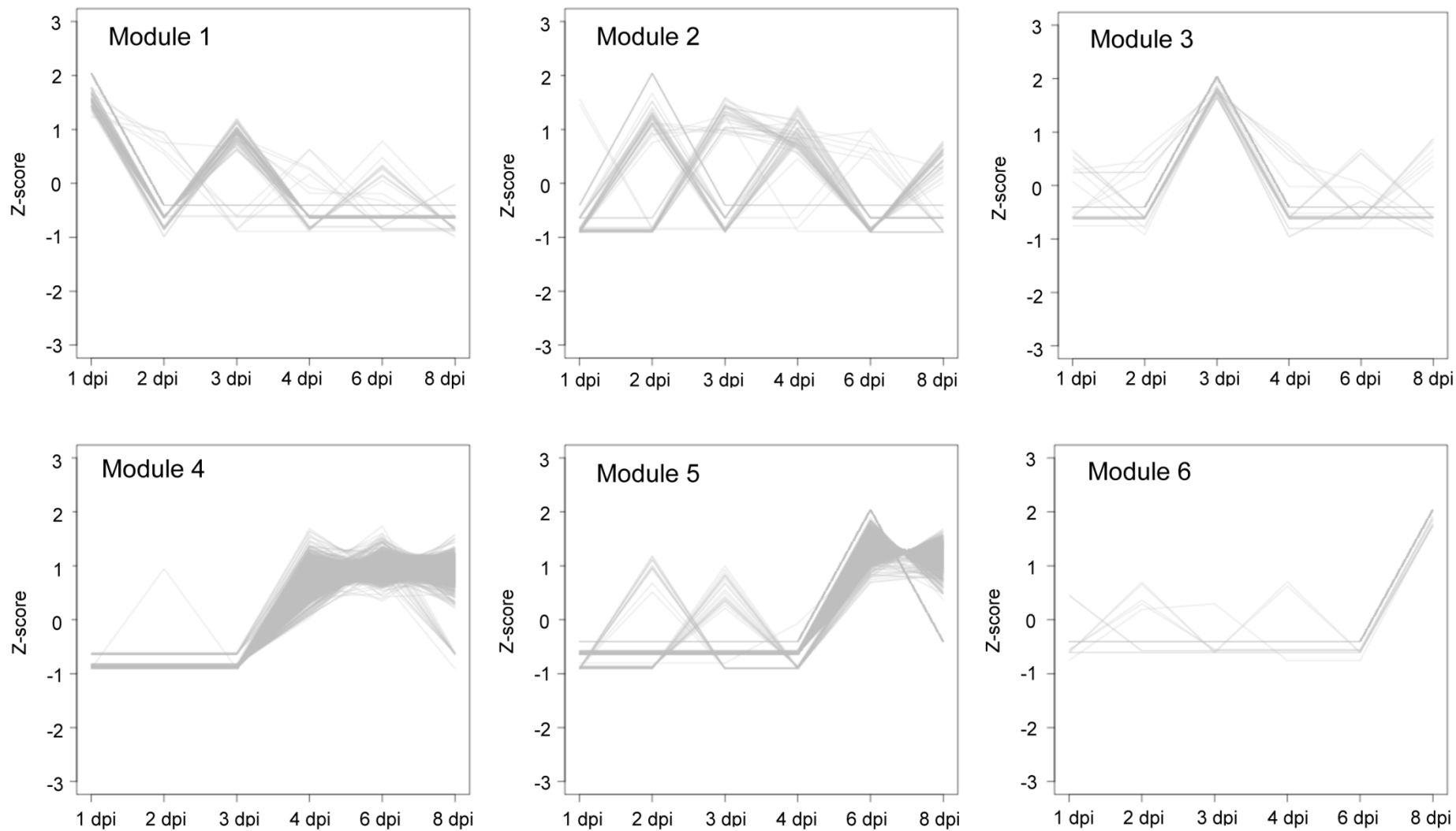

**Figure S3, Savory et al.**

Supplement: Figure S3 — Trend plots of the normalized gene expression values for each gene from six identified gene co-expression modules. Modules consisting of genes expressed modules 1, 2, 3, 4, 5, and 6 are shown. (PDF) [file pone.0035796.s003.pdf]
